# Supplementary material for: Peptostreptococcus anaerobius mediates anti-PD1 therapy resistance and exacerbates colorectal cancer via myeloid-derived suppressor cells in mice
Source: Nat Microbiol. 2024 May 15;9(6):1467–82. doi: 10.1038/s41564-024-01695-w (PMC11153135; doi:10.1038/s41564-024-01695-w)

nature microbiology

Article

https://doi.org/10.1038/s41564-024-01695-w

Peptostreptococcus anaerobius mediates anti-PD1 therapy resistance and exacerbates colorectal cancer via myeloid-derived suppressor cells in mice

In the format provided by the authors and unedited

Supplementary Table 1 Antibodies used for Western-blot, Immunofluorescence staining and Flow cytometry in this Experiments.

Antibodies	Source	Identifier	Dilution
**Anti-Integrin alpha 2**	abcam	ab133557	1:1000
**Anti-Integrin beta 1**	abcam	ab179471	1:1000
**Anti-NF-κB p65 (D14E12)**	cell signaling technology	8242S	1:1000
**Anti-Phospho-NF-κB p65 (Ser536)**	cell signaling technology	3033S	1:1000
**Anti-Beta-Actin (13E5)**	cell signaling technology	4970S	1:1000
**Anti-slamf4 (D5J9D)**	cell signaling technology	54560	1:1000 (wb)<br>1:100 (pull down)
**Anti-CD11b**	abcam	ab133357	1:200
**Alexa Fluor 555 Donkey anti-Mouse IgG (H+L)**	thermofisher	A-31570	1:500
**Alexa Fluor® 488 AffiniPure Goat Anti-Rabbit IgG (H+L)**	Jackson Immuno	111-545-003 (X2)	1:500
**normal mouse IgG**	Santa Cruz	sc-2025	1:200
**Brilliant Violet 605 anti-mouse CD45**	biolegend	103140	1:100
**Brilliant Violet 711 anti-mouse CD46**	biolegend	103147	1:100
**FITC anti-mouse CD3**	eBioscience	11-0031-85	1:100
**PE anti-mouse CD3**	biolegend	100206	1:100
**FITC anti-mouse CD4**	biolegend	100510	1:100
**PE-Cyanine5 anti-mouse CD4**	biolegend	100409	1:100
**Brilliant Violet 421 anti-mouse CD8**	biolegend	100738	1:100
**FITC anti-mouse IFNγ**	biolegend	505806	1:100
**PE-Cyanine5 anti-mouse TNF-α**	biolegend	506324	1:100
**FITC anti-mouse Gr-1**	biolegend	108405	1:100
**PE-Cyanine7 anti-mouse ly6g**	biolegend	127618	1:100
**PE-Cyanine7 anti-mouse Granzyme B**	biolegend	396411	1:100
**PE-Cyanine7 anti-mouse Arg1**	eBioscience	25-3697-80	1:100
**PI**	eBioscience	BMS500PI	1:100
**Brilliant Violet 421 anti-mouse PD-1 (CD279)**	biolegend	135221	1:100
**Brilliant Violet 421 anti-mouse Ly6C**	biolegend	128032	1:100
**purified anti-mouse CD16/32**	biolegend	101302	1:100
**PE anti-mouse iNOS**	biolegend	696806	1:100
**Alexa Fluor® 594 anti-mouse Ly-6G/Ly-6C (Gr-1)**	biolegend	108448	1:50 (IF)<br>1:100 (FC)
**PE-Cyanine5 anti-mouse CD11b**	biolegend	101210	1:100
**FITC anti-mouse CD12b**	biolegend	101206	1:100

Supplementary Table 2 Chemicals, Recombinant Proteins and Kits used in this Experiments

Chemicals and kits	Sources	Identifier
**Azoxymethane**	Sigma-Aldrich	A5486
**Dextran Sodium Sulfate**	MP Biomedicals	MFCD00081551
**Corning matrigel matrix**	Corning	356231
**Recombinant mouse GM-CSF**	biolegend	576304
**Recombinant mouse IL-6**	biolegend	575704
**CFSE**	thermofisher	C1157
**Dnase I, Grade II**	Roche	10104159001
**Collagenase IV**	Roche	58001
**InVivoPlus anti-mouse Ly6G**	Bio XCell	BP0075-1
**InVivoMab anti-mouse PD-1**	Bio XCell	BE0146
**Human Slamf4**	Abclonal	RP00167
**IC Fixation Buffer 125 mL**	eBiosciences	00-8822-49
**Permeabilization Buffer 10X**	Invitrogen	00-8333-56
**RBC Lysis Buffer (10X)**	biolegend	420301
**Recombinant mouse IL-2**	biolegend	575404
**Ionomycin**	STEMCELL Technologies	73724
**phorbol 12-myristate 13-acetate**	abcam	ab120297
**Human CXCL1 Elisa kit**	abcam	ab190805
**Mouse CXCL1 Elisa kit**	abcam	ab216951
**Human Chemokine Antibody Array**	abcam	ab169812
**Mouse MDSC (CD11b+Gr1+) Isolation Kit**	STEMCELL Technologies	19867
**Mouse T Cell Isolation Kit**	STEMCELL Technologies	19851

Supplementary Table 3 Primers used for q-PCR

Gene	Oligonucleotides
**mouse-b-actin-F**	TGTTACCAACTGGGACGACATG
**mouse-b-actin-R**	CTGGATGGCTACGTACATGGCT
**mouse-Slamf1-F**	CAGAAATCAGGGCCTCAAGAG
**mouse-Slamf1-R**	CACTGGCATAAACTGTGGTGG
**mouse-Slamf2-F**	CCCAAGCCTTCCATAGAAATCAA
**mouse-Slamf2-R**	CCAAGTATAGTCAACATGCTGGT
**mouse-Slamf3-F**	TCAGGGATGCTAGGGGGTTC
**mouse-Slamf3-R**	TTCGCTGACTTTGAGTCTGCC
**mouse-Slamf4-F**	CTCGGGGCCATCATTTGTTTC
**mouse-Slamf4-R**	GCTAGAAGGGAGCTGAACATCA
**mouse-Slamf6-F**	TATGCACAAGTCACTCGTCCA
**mouse-Slamf6-R**	GGTTATAGCCGGTTAAAGCCAC
**mouse-Tlr2-F**	GCAAACGCTGTTCTGCTCAG
**mouse-Tlr2-R**	AGGCGTCTCCCTCTATTGTATT
**mouse-Tlr4-F**	ATGGCATGGCTTACACCACC
**mouse-Tlr4-R**	GAGGCCAATTTTGTCTCCACA
**mouse-Tlr5-F**	GCAGGATCATGGCATGTCAAC
**mouse-Tlr5-R**	ATCTGGGTGAGGTTACAGCCT
**mouse-Tlr6-F**	TGAGCCAAGACAGAAAACCCA
**mouse-Tlr6-R**	GGGACATGAGTAAGGTTCCTGTT
**mouse-Tlr9-F**	AATCCCTCATATCCCTGTCCC
**mouse-Tlr9-R**	GTTGCCGTCCATGAATAGGAAG
**mouse-Cxcl1-F**	CTGGGATTCACCTCAAGAACATC
**mouse-Cxcl1-R**	CAGGGTCAAGGCAAGCCTC
**mouse-Arg1-F**	CTCCAAGCCAAAGTCCTTAGAG
**mouse-Arg1-R**	AGGAGCTGTCATTAGGGACATC
**mouse-inos-F**	GGGCTGTCACGGAGATCA
**mouse-inos-R**	CCATGATGGTCACATTCTGC
**P. anaerobius -F**	CTGATTTGATGCTTGCATTA
**P. anaerobius-R**	AGCCCCGAAGGGAAGGTGTG

Supplementary Table 4 Mass Spectrometry Results of Bands Identified from 50-100kDa fraction from culture supernatant of *P. anaerobius*

Protein Group	Accession	Coverage(%)	Area	#Unique	#Spec	Mass	Description
1	A0A379CGB7|A0A379CGB7_9FIRM	92.98	60856443000	105	2856	80.864	N-acetylmuramoyl-L-alanine amidase LytC OS=Peptostreptococcus anaerobius OX=1261 GN=lytC_22 PE=4 SV=1
10	A0A379CFM5|A0A379CFM5_9FIRM	50	100711096	33	46	81.972	N-acetylmuramoyl-L-alanine amidase LytC OS=Peptostreptococcus anaerobius OX=1261 GN=lytC_21 PE=4 SV=1
151	A0A6N7X0K1|A0A6N7X0K1_9FIRM	48.12	5657323	2	2	14.493	30S ribosomal protein S11 OS=Peptostreptococcus anaerobius OX=1261 GN=rpsK PE=3 SV=1
151	A0A135YUR7|A0A135YUR7_9FIRM	48.12	5657323	2	2	14.479.	30S ribosomal protein S11 OS=Peptostreptococcus anaerobius OX=1261 GN=rpsK PE=3 SV=1
151	D3MTE5|D3MTE5_9FIRM	48.12	5657323	2	2	14.479	30S ribosomal protein S11 OS=Peptostreptococcus anaerobius 653-L OX=596329 GN=rpsK PE=3 SV=1
23	A0A379CHV1|A0A379CHV1_9FIRM	41.11	57788176	19	24	67.248	Uncharacterized peptidase SA1530 OS=Peptostreptococcus anaerobius OX=1261 GN=NCTC11460_01965 PE=4 SV=1
28	A0A379CFM8|A0A379CFM8_9FIRM	40.31	42666476	16	20	62.369	Pyruvate kinase OS=Peptostreptococcus anaerobius OX=1261 GN=pyk PE=3 SV=1
28	D3MPK9|D3MPK9_9FIRM	40.31	42666476	16	20	62.392	Pyruvate kinase OS=Peptostreptococcus anaerobius 653-L OX=596329 GN=pyk PE=3 SV=1
28	A0A135YSW5|A0A135YSW5_9FIRM	40.31	42666476	16	20	62.366	Pyruvate kinase OS=Peptostreptococcus anaerobius OX=1261 GN=HMPREF3195_01071 PE=3 SV=1
2	D3MU98|D3MU98_9FIRM	38.11	629723580	8	951	80.903	Putative cell wall binding repeat 2 OS=Peptostreptococcus anaerobius 653-L OX=596329 GN=HMPREF0631_1404 PE=4 SV=1
7	A0A379CFB2|A0A379CFB2_9FIRM	33.3	51208652	25	34	120.494	N-acetylmuramoyl-L-alanine amidase LytC OS=Peptostreptococcus anaerobius OX=1261 GN=lytC_20 PE=4 SV=1
570	A0A379CEQ2|A0A379CEQ2_9FIRM	30.99	1518149.2	1	1	84.16	Barstar (Barnase inhibitor) OS=Peptostreptococcus anaerobius OX=1261 GN=NCTC11460_00474 PE=3 SV=1
40	A0A379CF33|A0A379CF33_9FIRM	24.01	23831450	11	15	81.537	N-acetylmuramoyl-L-alanine amidase LytC OS=Peptostreptococcus anaerobius OX=1261 GN=lytC_11 PE=4 SV=1
43	A0A379CHG0|A0A379CHG0_9FIRM	23.21	16648673	9	15	71.305	85 kDa vitronectin-binding protein OS=Peptostreptococcus anaerobius OX=1261 GN=fusA_2 PE=3 SV=1

Supplementary Table 5 siRNA Used in the Experiments.

Gene	Species	Company	Identifier
siCXCL1-1	Human	Invitrogen	s6215
siCXCL1-2	Human	Invitrogen	s6216
siCXCL3-1	Human	Invitrogen	s6221
siCXCL3-1	Human	Invitrogen	s6223
siITGA2	Human	Invitrogen	s7573
siITGB1	Human	GCACCAGCCCAUUUAGCUATT	

Fig 5a

MDSC Anti-actin

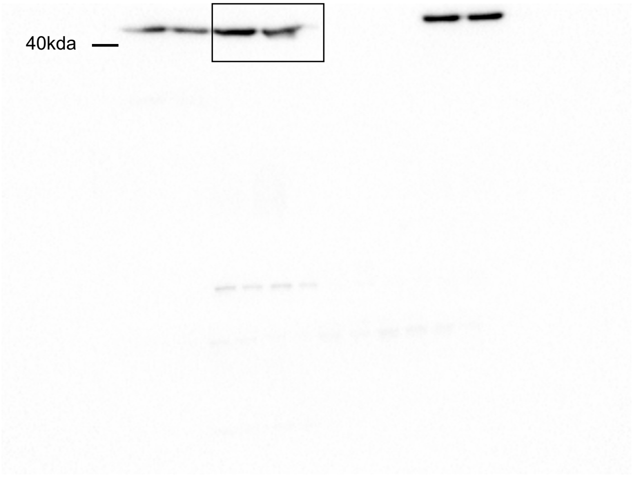

Anti-Slamf4

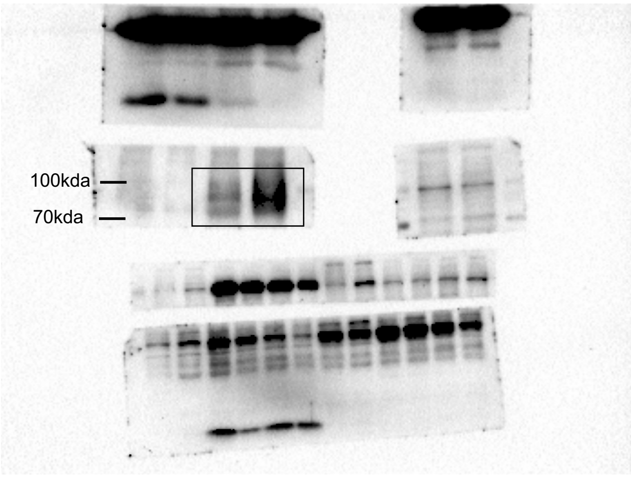

Fig 5f

Anti-GST

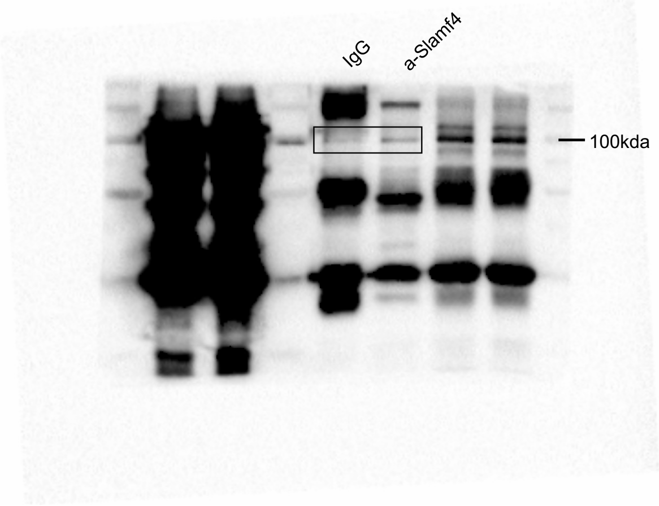

Anti-Slamf4

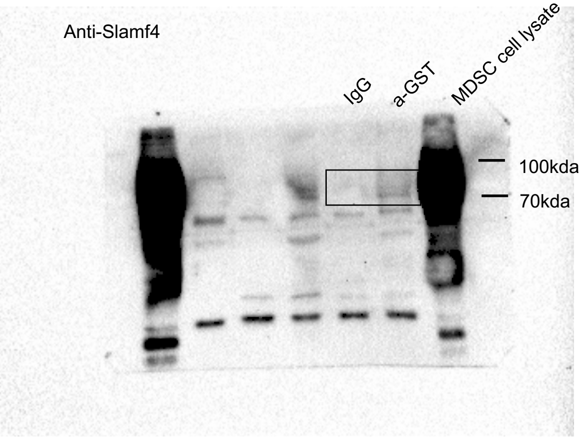

Fig 5f

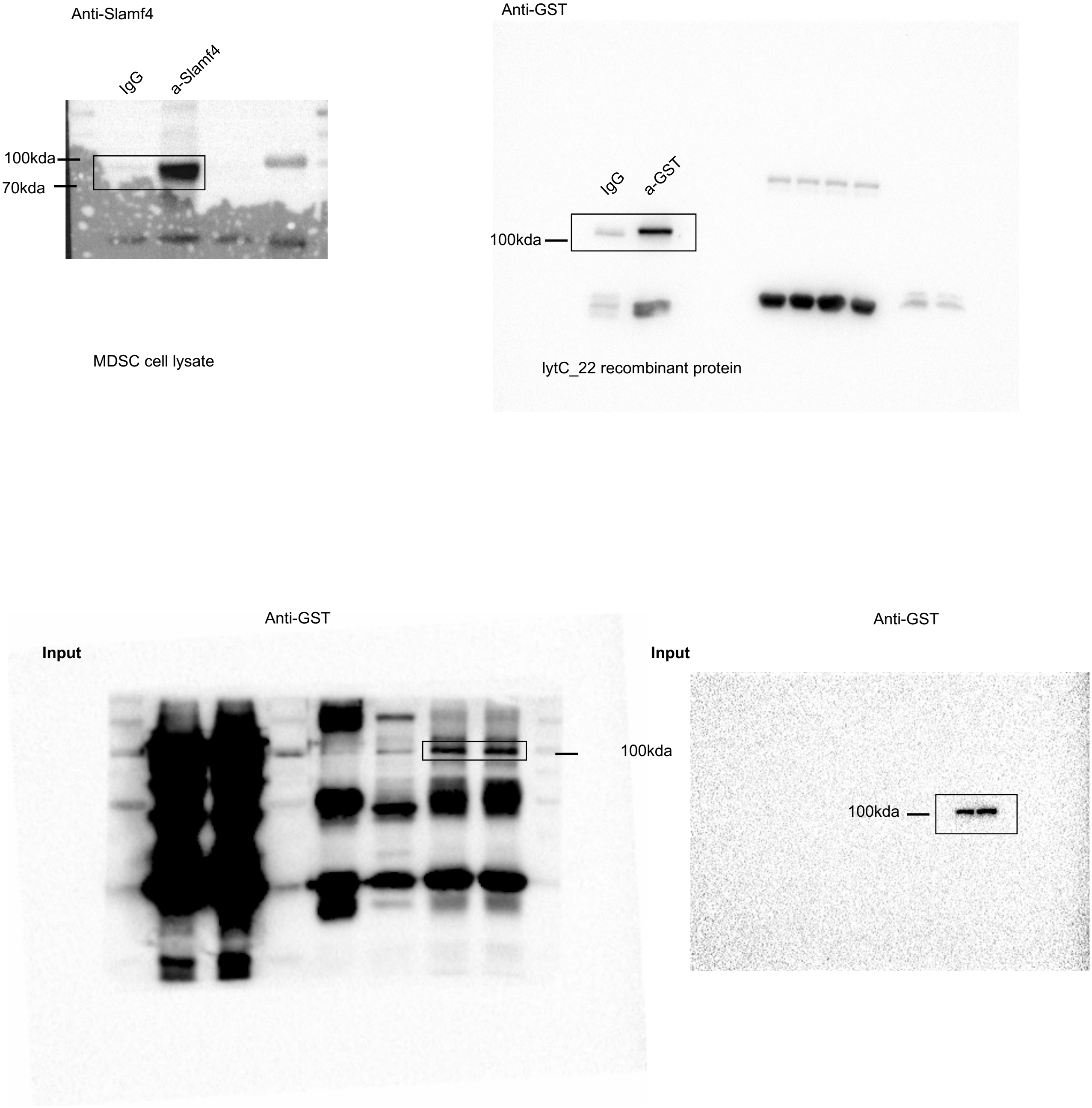
Anti-Slamf4
IgG
a-Slamf4
100kda
70kda
MDSC cell lysate
Anti-GST
IgG
a-GST
100kda
lytC_22 recombinant protein
Anti-GST
Input
100kda
Anti-GST
Input
100kda

Fig 5f

Anti-Slamf4

Input

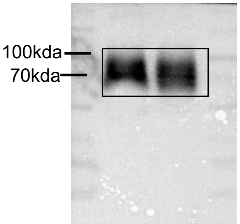

MDSC cell lysate

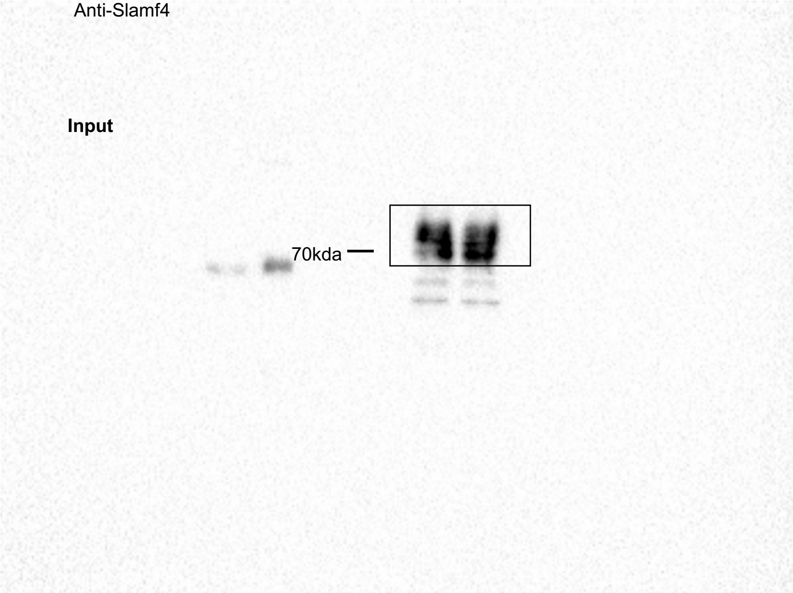

Fig 5g

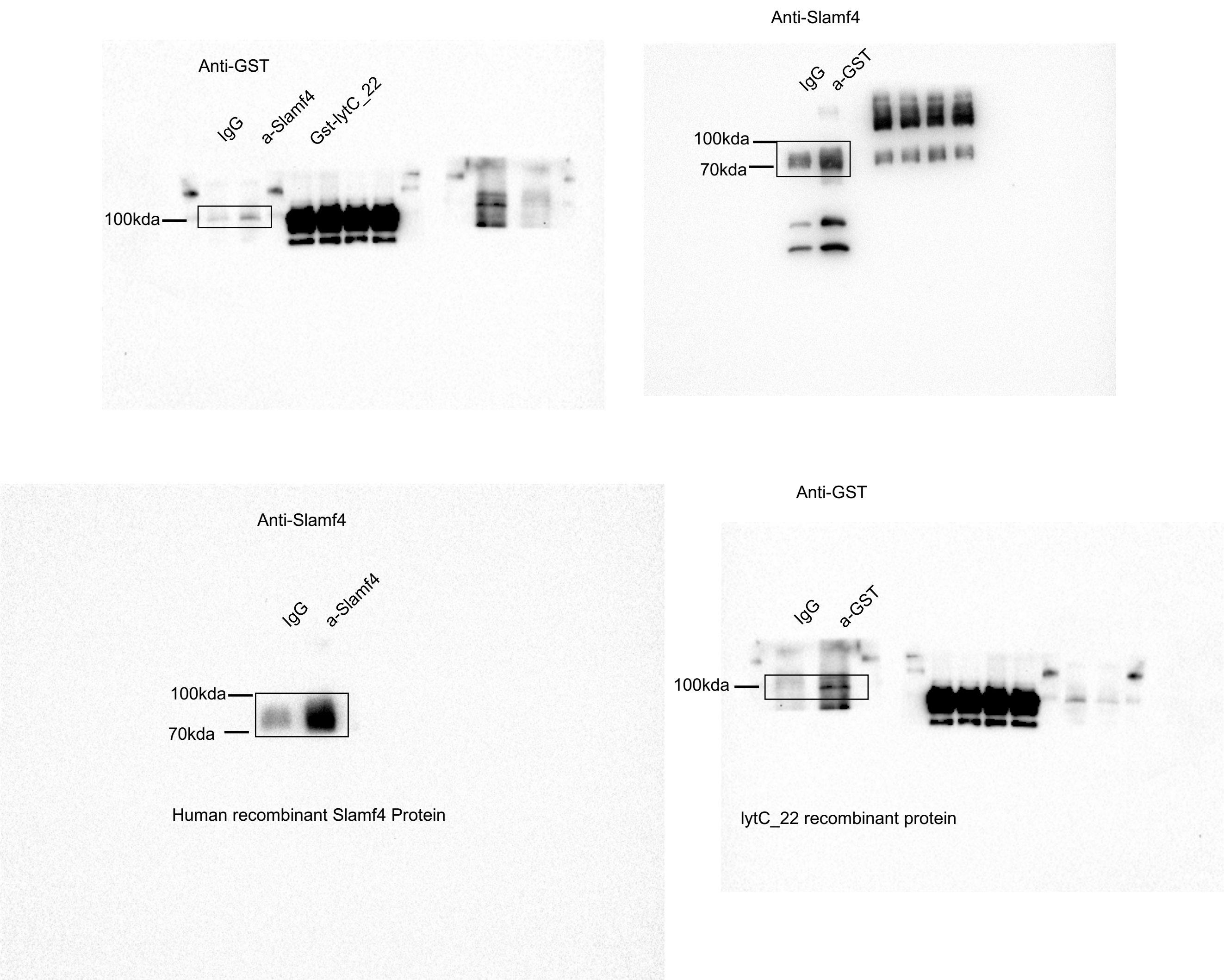
Anti-GST
IgG
a-Slamf4
Gst-lytC_22
100kda
Anti-Slamf4
IgG
a-GST
100kda
70kda
Anti-Slamf4
IgG
a-Slamf4
100kda
70kda
Human recombinant Slamf4 Protein
Anti-GST
IgG
a-GST
100kda
lytC_22 recombinant protein

Fig 5g

Input

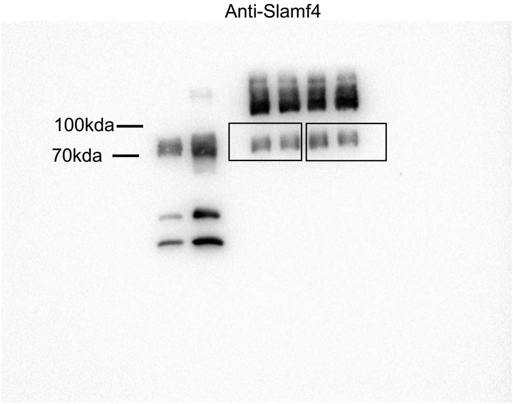

Input

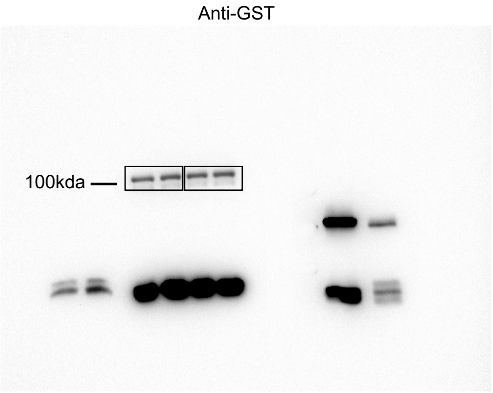

Fig 6b

Anti-Slamf4

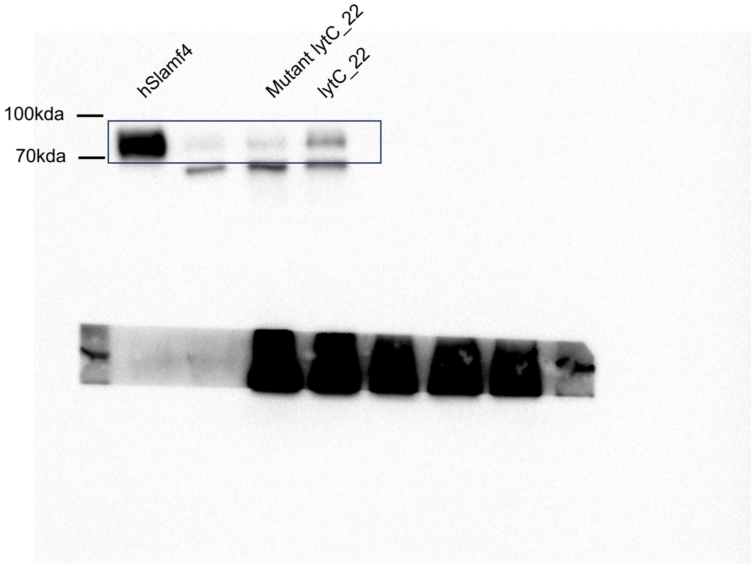

Input

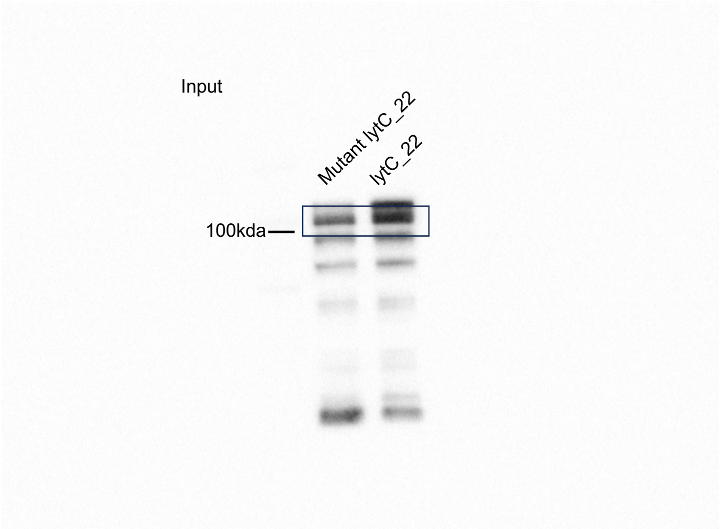

Extended Figure 5a

HCT116

HCT116 Actin

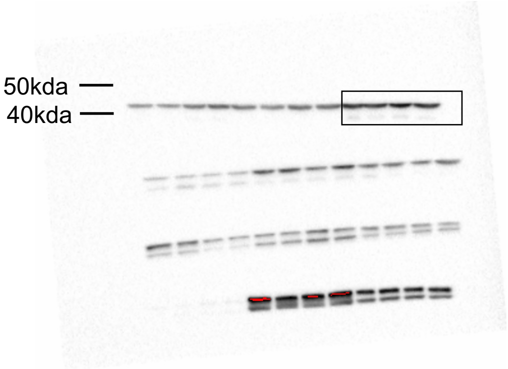

HCT116 NF-KB p65

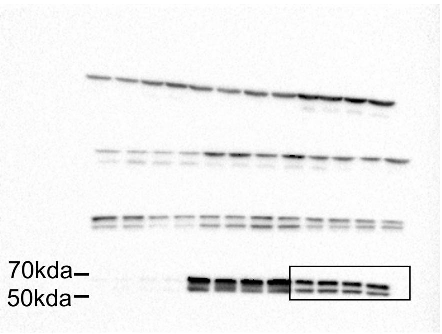

HCT116 p-NF-KB p65

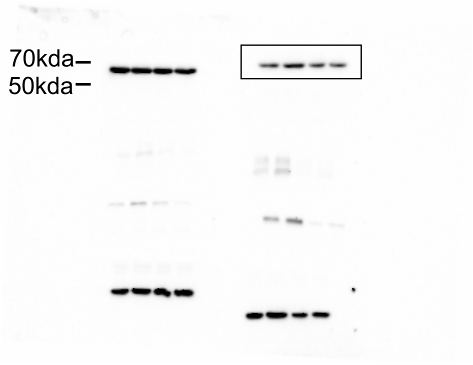

HCT116 Integrin $\beta 1$

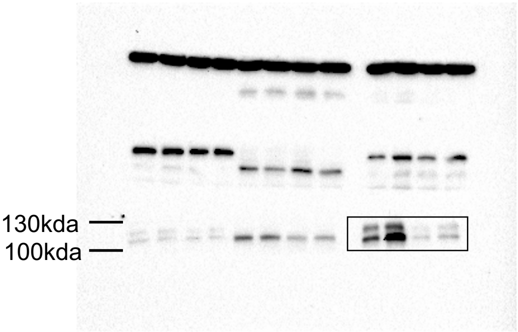

HCT116 Integrin $\alpha 2$

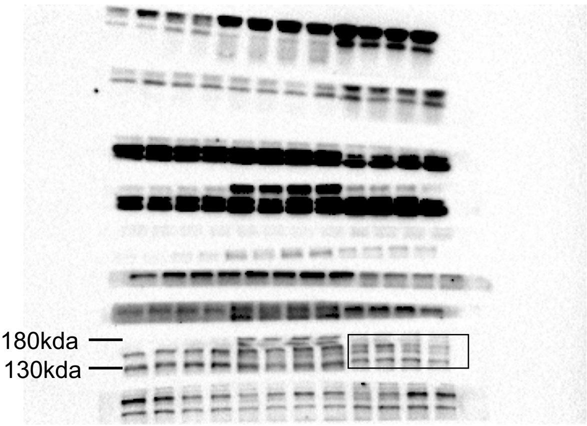

Extended Figure 5a

Caco-2 actin

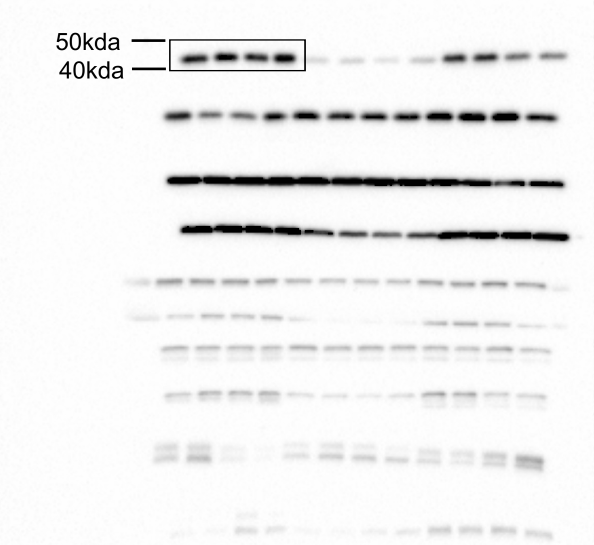

Caco-2 NF-KB p65

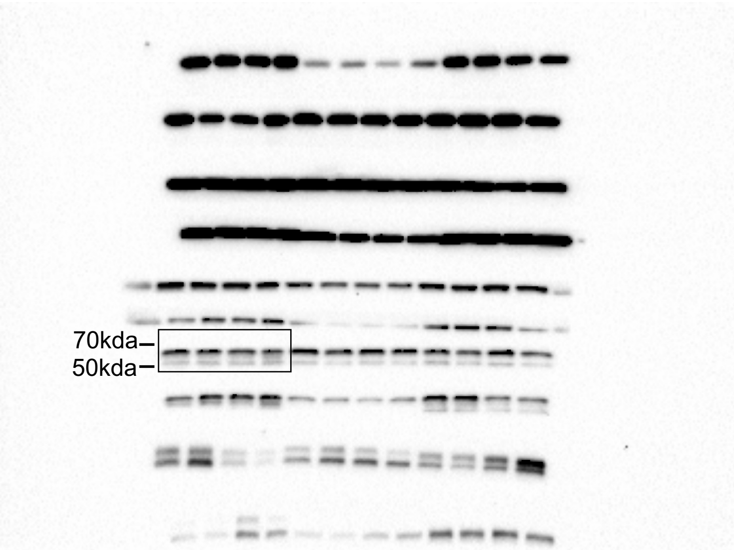

Caco-2 p-NF-KB p65

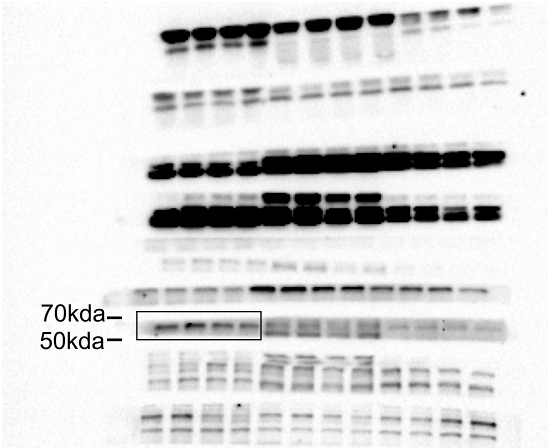

Caco-2 Integrin $\beta 1$

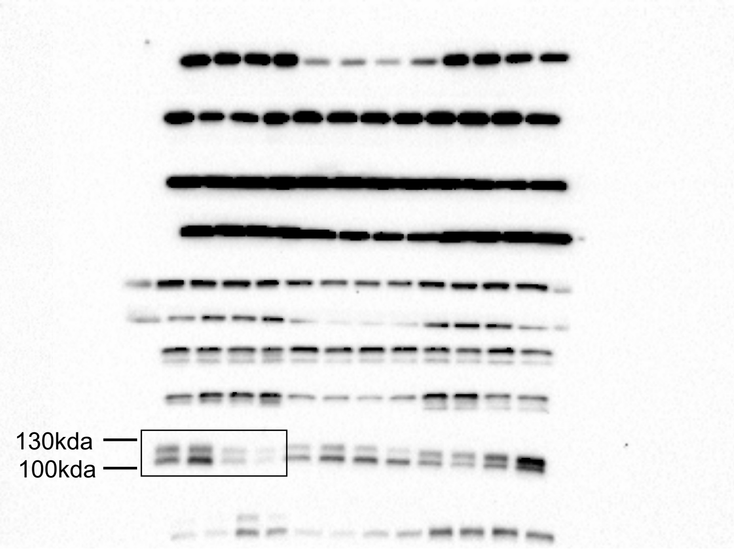

Caco-2 Integrin $\alpha 2$

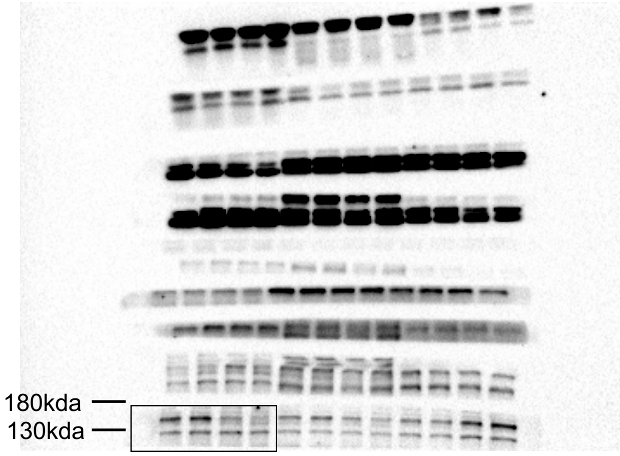

Extended Figure 5b

HCT116 actin

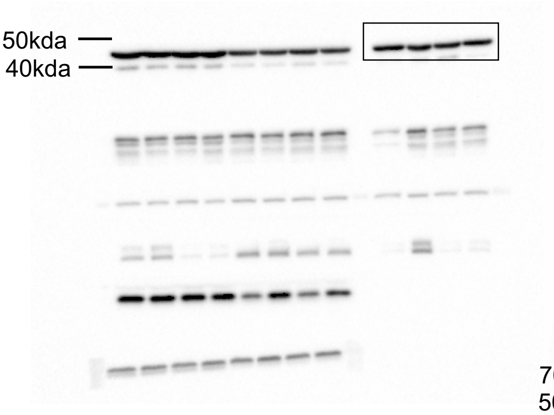

HCT116 NF-KB p65

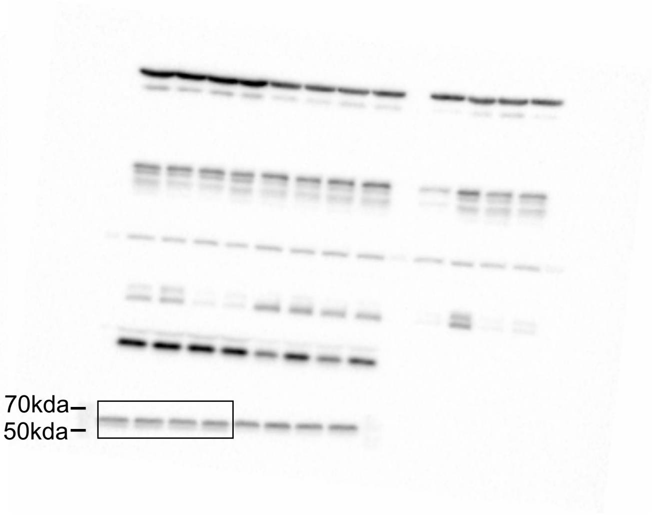

HCT116 p-NF-KB p65

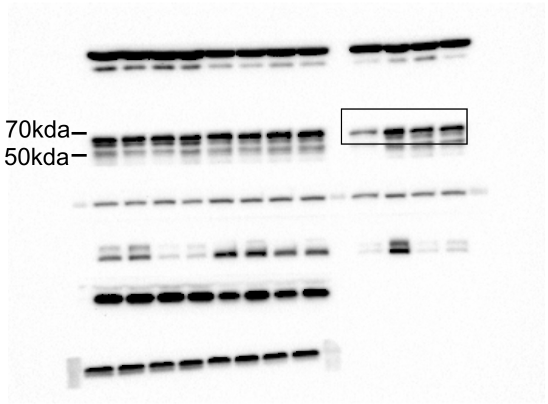

HCT116 Integrin $\beta 1$

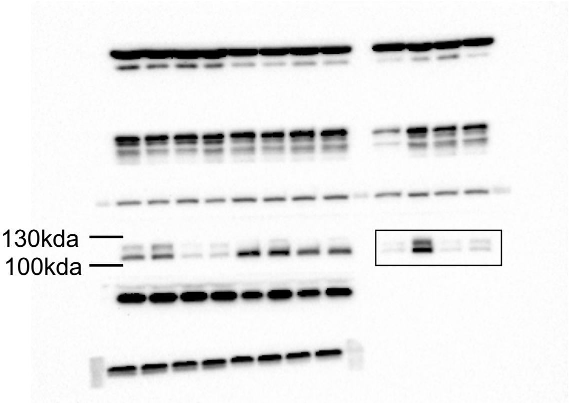

HCT116 Integrin $\alpha 2$

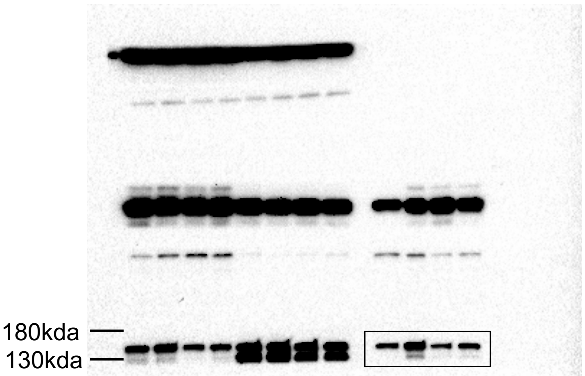

Extended Figure 5b

MC38 actin

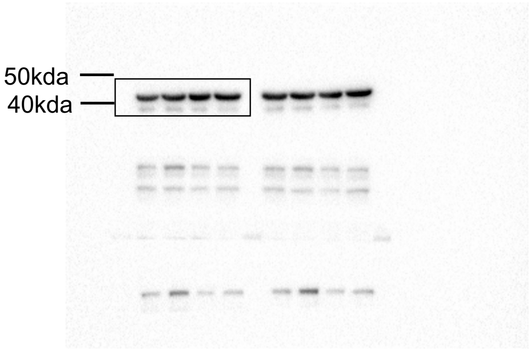

MC38 NF-KB p65

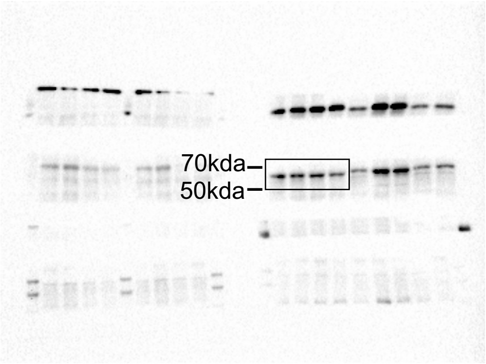

MC38 p-NF-KB p65

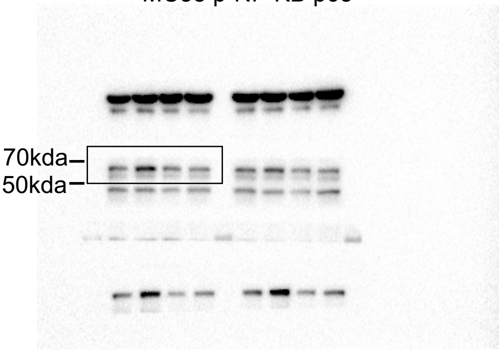

MC38 Integrin $\beta 1$

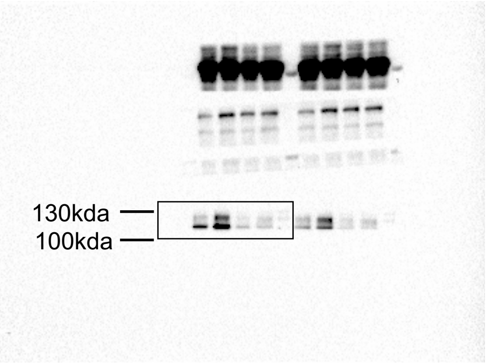

MC38 Integrin $\alpha 2$

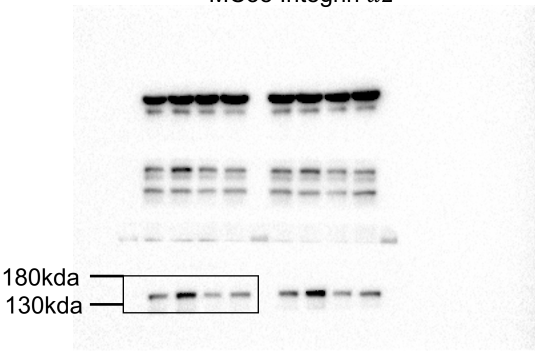

Extended Figure 5c

HCT116 Cyto-Actin

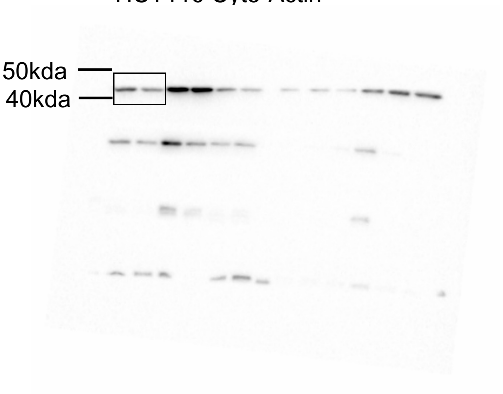

HCT116 Cyto-p-NF-KB p65

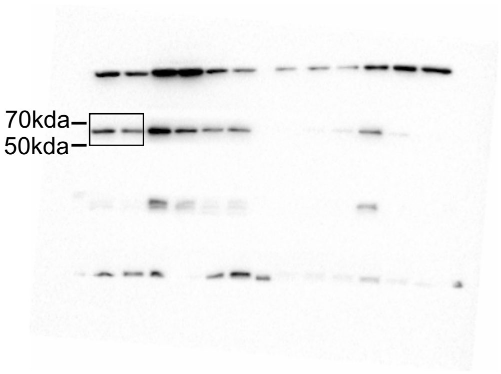

HCT116 Cyto-NF-KB p65

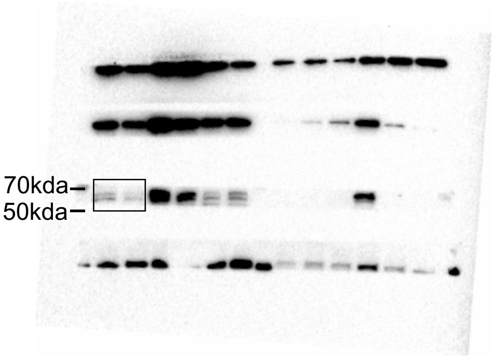

Caco-2 Cyto-Actin

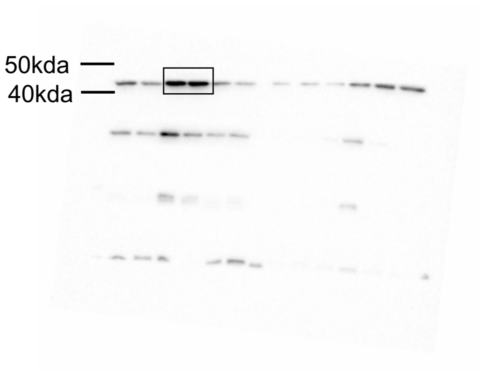

Caco-2 Cyto-p-NF-KB p65

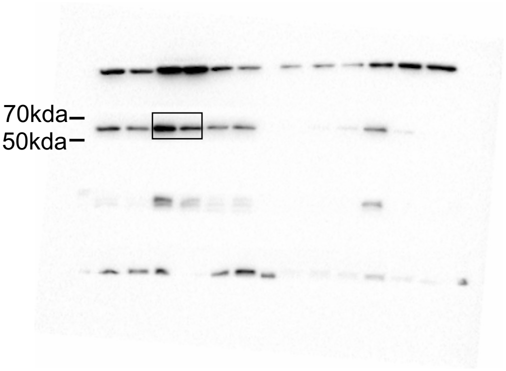

Caco-2 Cyto-NF-KB p65

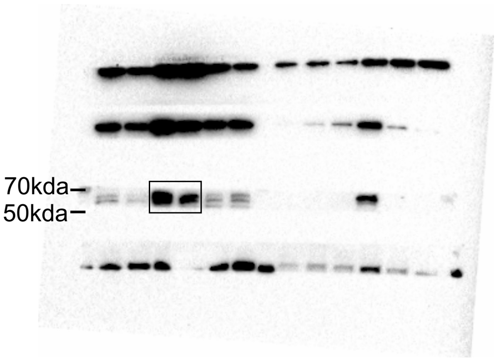

Extended Figure 5c

MC38 Cyto-Actin

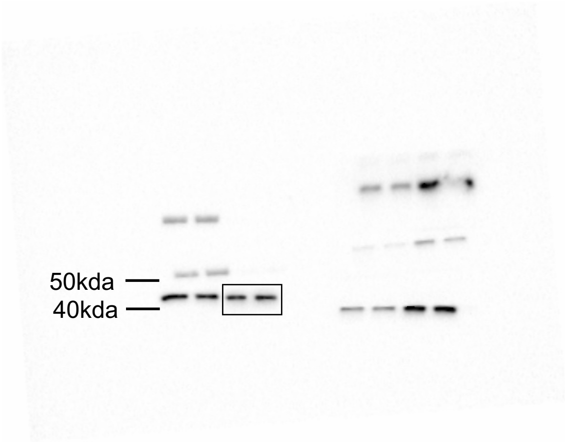

MC38 Cyto-NF-KB
p65

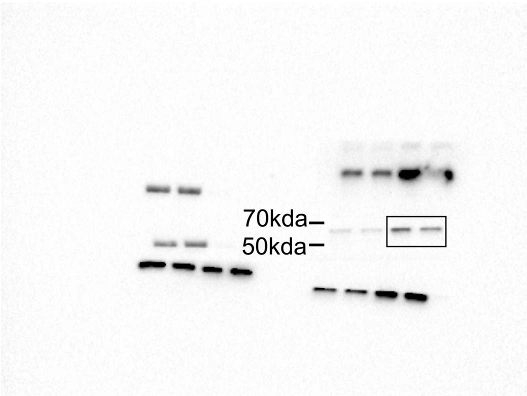

MC38 Cyto-p-NF-KB p65

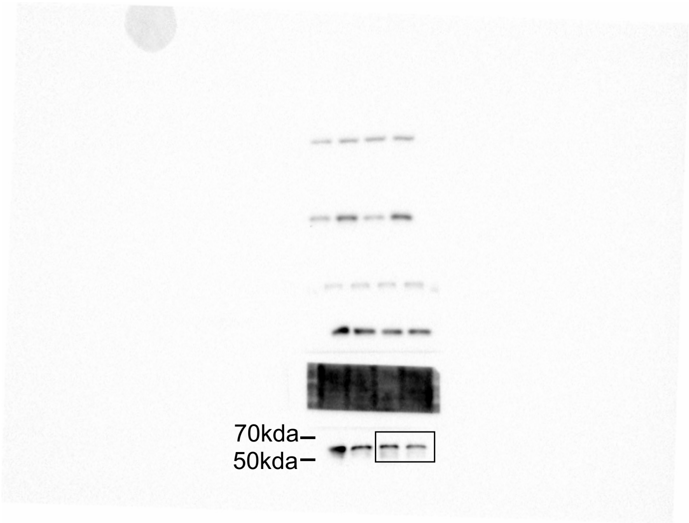

Extended Figure 5c

HCT116 nu-Lamin A/C

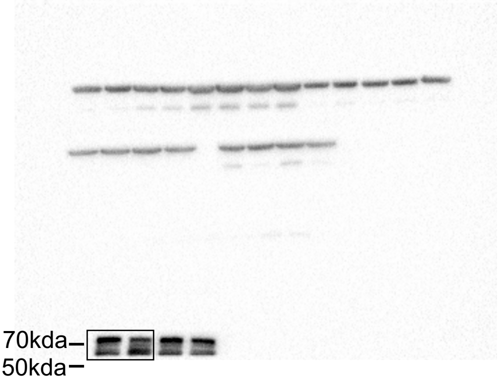

HCT116 nu-NF-KB p65

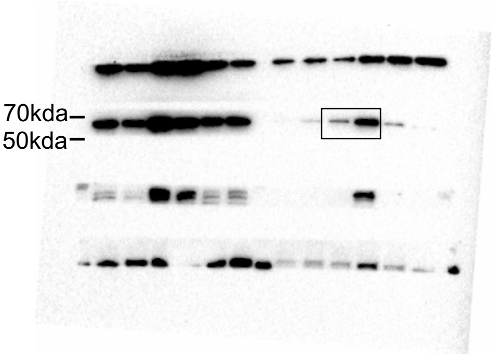

HCT116 nu-p-NF-KB p65

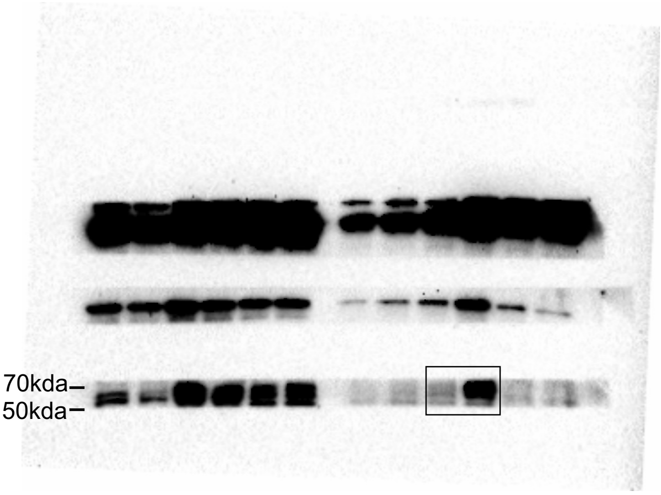

Caco-2 nu-Lamin A/C

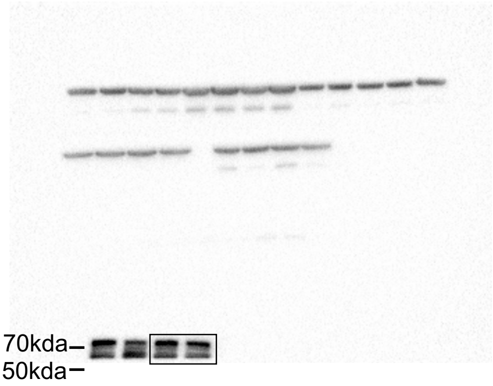

Caco-2 nu-NF-KB p65

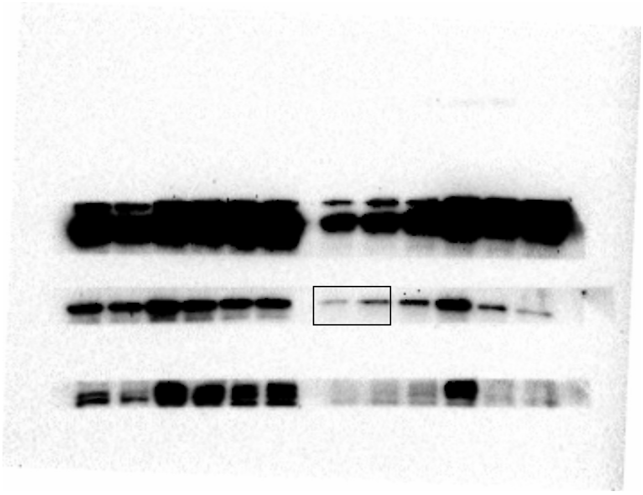

Caco-2 nu-p-NF-KB p65

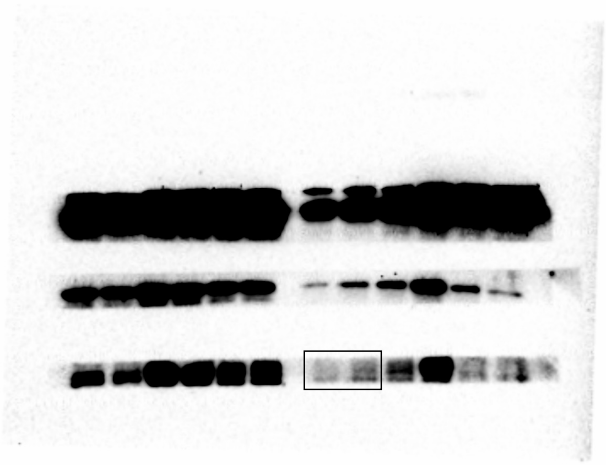

Extended Figure 5c

MC38 nu-lamin A/C

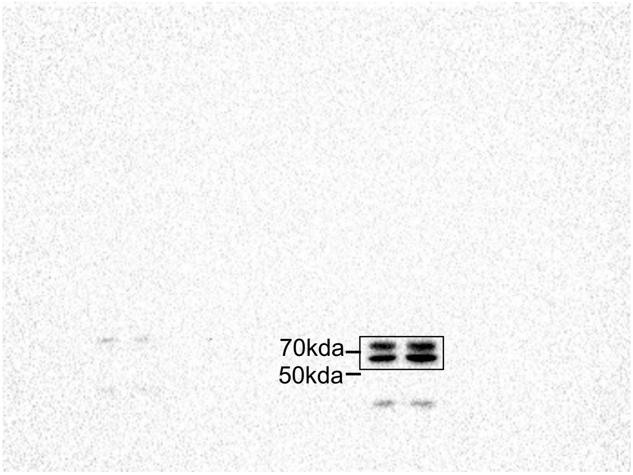

MC38 nu-NF-KB
p65

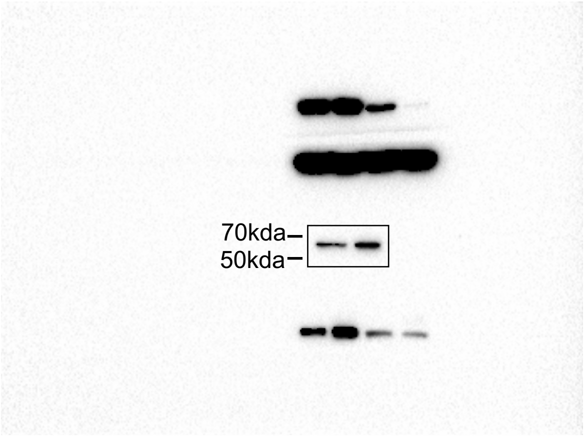

MC38 nu-p-NF-KB p65

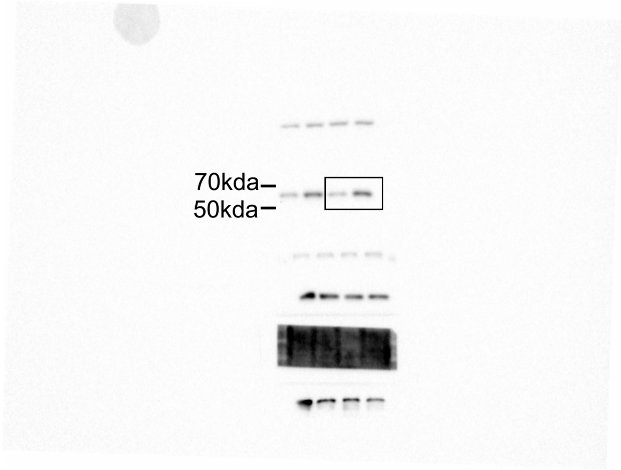

Extended Figure 5d

HCT116 actin

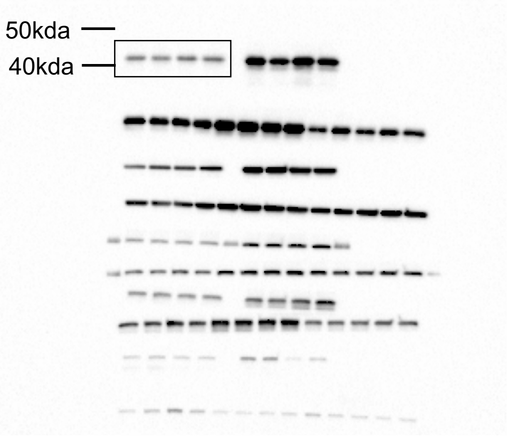

HCT116 NF-KB p65

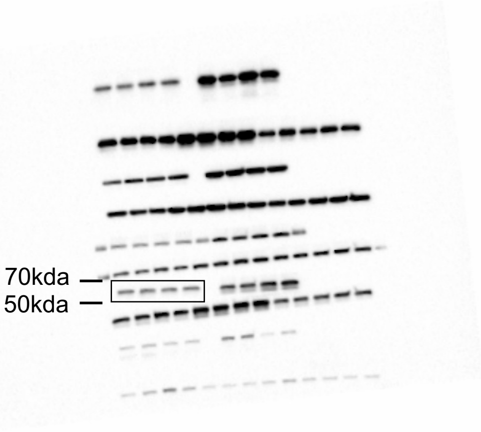

HCT116 p-NF-KB p65

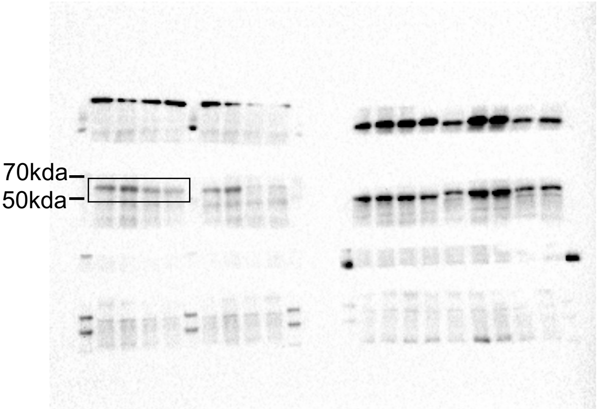

Caco-2 actin

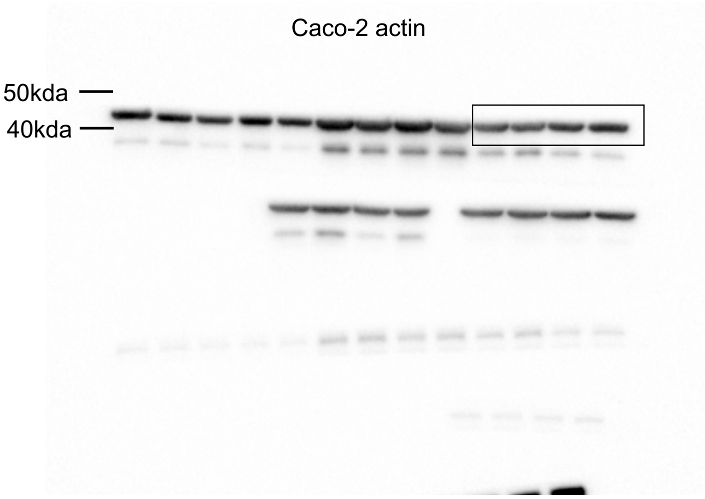

Caco-2 NF-KB p65

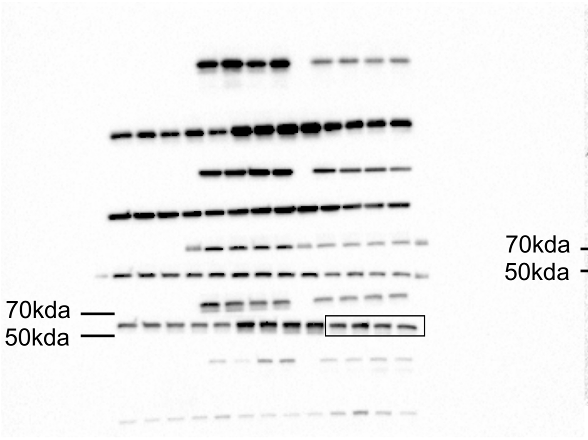

Caco-2 p-NF-KB p65

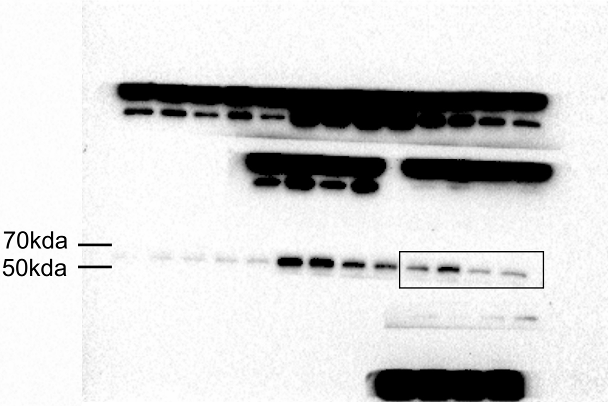

Extended Figure 5d

MC38 actin

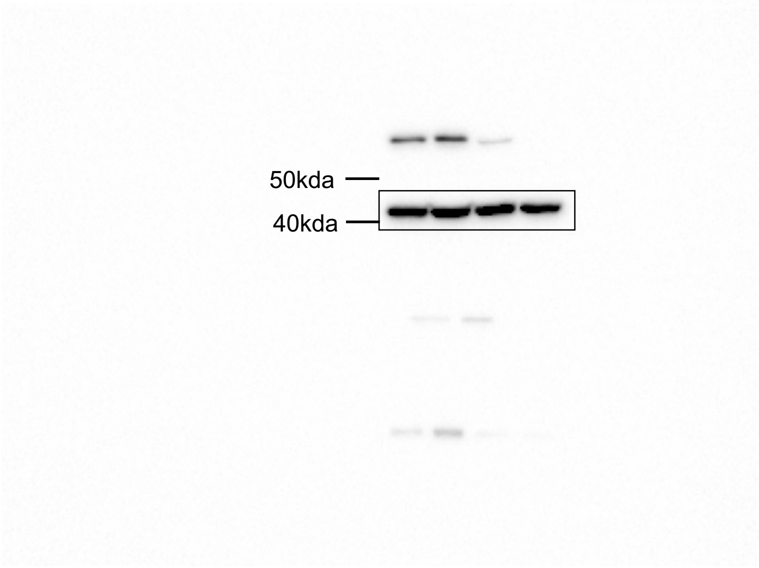

MC38 NF-KB p65

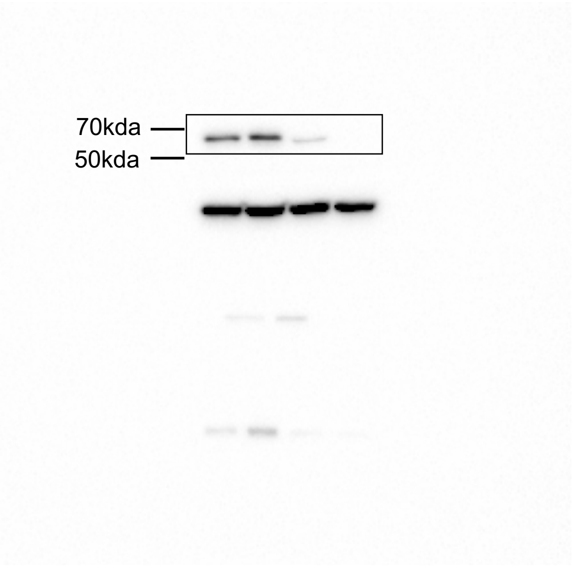

MC38 p-NF-KB p65

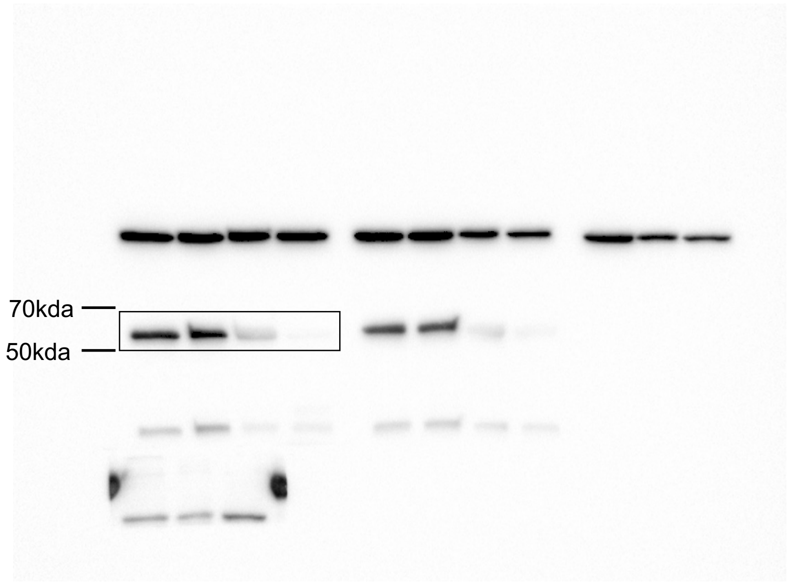

Extended Figure 5k

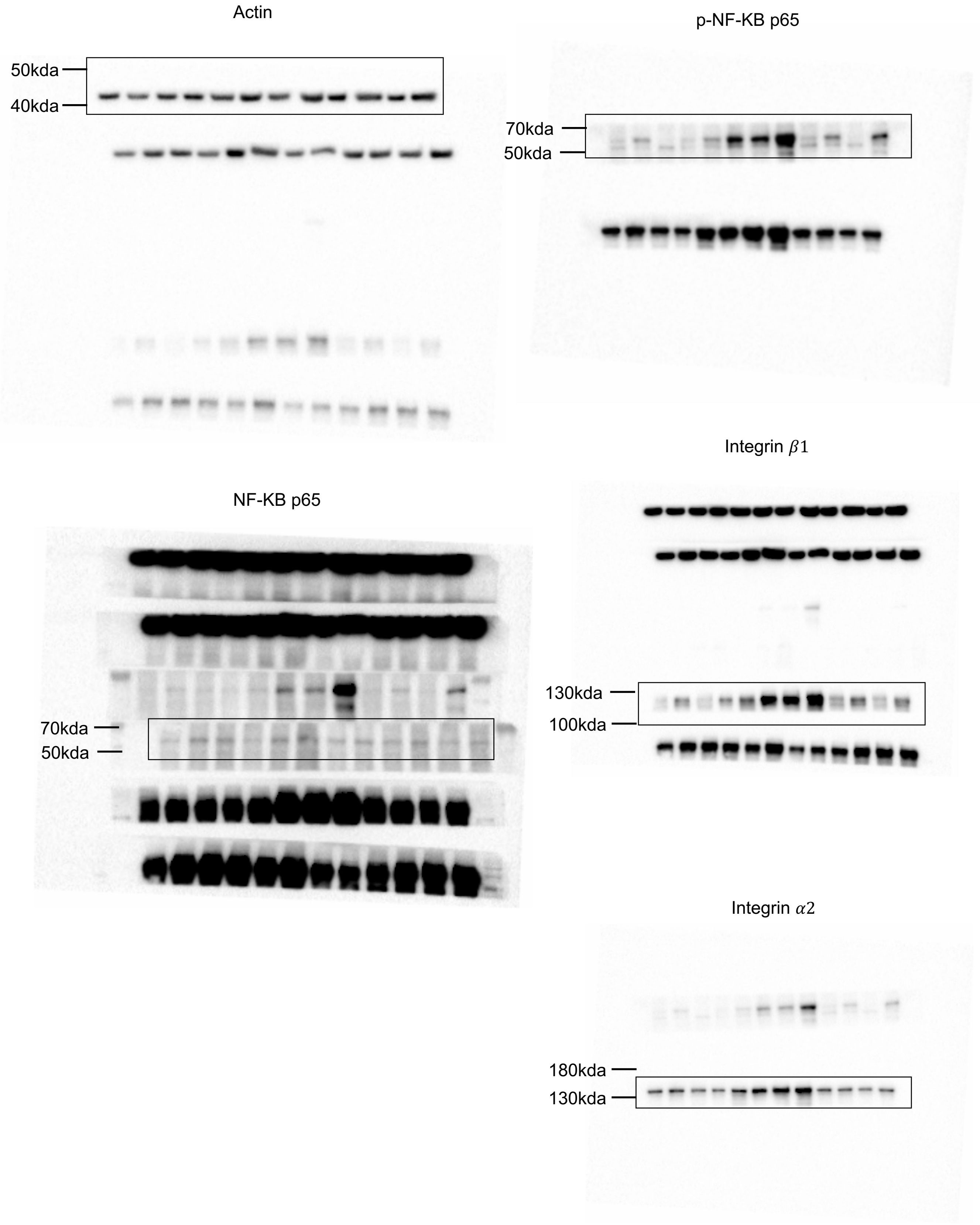

Extended Data Fig 7a

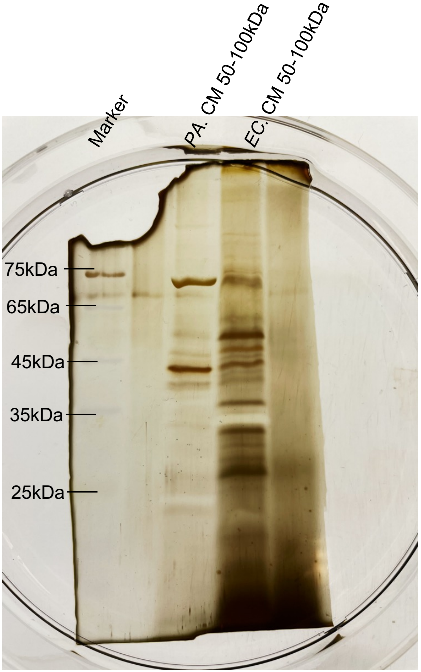

Extended Data Fig 8g

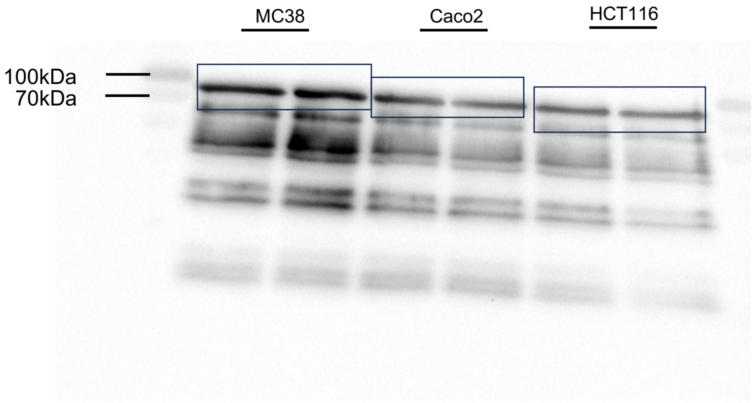

Supplement: Supplementary file 1 — Supplementary Tables 1–5, Supplementary Figs. 1–5 and unprocessed scans of blots and gels. [file 41564_2024_1695_MOESM1_ESM.pdf]
