# Supplementary material for: Peptostreptococcus anaerobius mediates anti-PD1 therapy resistance and exacerbates colorectal cancer via myeloid-derived suppressor cells in mice
Source: Nat Microbiol. 2024 May 15;9(6):1467–82. doi: 10.1038/s41564-024-01695-w (PMC11153135; doi:10.1038/s41564-024-01695-w)
Supplement: Supplementary file 2 — Reporting Summary [file 41564_2024_1695_MOESM2_ESM.pdf]

nature portfolio

Corresponding author(s): Jun Yu

Last updated by author(s): Jan 25, 2024

Reporting Summary

Nature Portfolio wishes to improve the reproducibility of the work that we publish. This form provides structure for consistency and transparency in reporting. For further information on Nature Portfolio policies, see our Editorial Policies and the Editorial Policy Checklist.

Statistics

For all statistical analyses, confirm that the following items are present in the figure legend, table legend, main text, or Methods section.

n/a	Confirmed	
☐	☒	The exact sample size (*n*) for each experimental group/condition, given as a discrete number and unit of measurement
☐	☒	A statement on whether measurements were taken from distinct samples or whether the same sample was measured repeatedly
☐	☒	The statistical test(s) used AND whether they are one- or two-sided<br>*Only common tests should be described solely by name; describe more complex techniques in the Methods section.*
☒	☐	A description of all covariates tested
☒	☐	A description of any assumptions or corrections, such as tests of normality and adjustment for multiple comparisons
☐	☒	A full description of the statistical parameters including central tendency (e.g. means) or other basic estimates (e.g. regression coefficient) AND variation (e.g. standard deviation) or associated estimates of uncertainty (e.g. confidence intervals)
☐	☒	For null hypothesis testing, the test statistic (e.g. *F*, *t*, *r*) with confidence intervals, effect sizes, degrees of freedom and *P* value noted<br>*Give P values as exact values whenever suitable.*
☒	☐	For Bayesian analysis, information on the choice of priors and Markov chain Monte Carlo settings
☒	☐	For hierarchical and complex designs, identification of the appropriate level for tests and full reporting of outcomes
☒	☐	Estimates of effect sizes (e.g. Cohen's *d*, Pearson's *r*), indicating how they were calculated

Our web collection on statistics for biologists contains articles on many of the points above.

Software and code

Policy information about availability of computer code

Data collection	Image Lab software v6.1.0 (Bio-rad), QuantStudio Real Time PCR software v1.7.2 (Applied Biosystems), FACS Diva 6.1.3 (BD Biosciences)
Data analysis	FlowJo (v9), Image J 1.51, Prism v9, HDOCK software, Pymol 2.1 software

For manuscripts utilizing custom algorithms or software that are central to the research but not yet described in published literature, software must be made available to editors and reviewers. We strongly encourage code deposition in a community repository (e.g. GitHub). See the Nature Portfolio guidelines for submitting code & software for further information.

Data

Policy information about availability of data

All manuscripts must include a data availability statement. This statement should provide the following information, where applicable:

- Accession codes, unique identifiers, or web links for publicly available datasets
- A description of any restrictions on data availability
- For clinical datasets or third party data, please ensure that the statement adheres to our policy

The datasets generated during and/or analysed during the current study are available from the corresponding authors upon request. All related raw data has been deposited to the public datasets with accession code.

Human research participants

Policy information about studies involving human research participants and Sex and Gender in Research.

Reporting on sex and gender	N/A
Population characteristics	N/A
Recruitment	N/A
Ethics oversight	N/A

Note that full information on the approval of the study protocol must also be provided in the manuscript.

Field-specific reporting

Please select the one below that is the best fit for your research. If you are not sure, read the appropriate sections before making your selection.

☒ Life sciences ☐ Behavioural & social sciences ☐ Ecological, evolutionary & environmental sciences

For a reference copy of the document with all sections, see nature.com/documents/nr-reporting-summary-flat.pdf

Life sciences study design

All studies must disclose on these points even when the disclosure is negative.

Sample size	No statistical methods were used to predetermine sample size, but our sample sizes are similar to those in previous reports
Data exclusions	No exclusion of data was performed
Replication	All experiments were repeated 3 three times as three independent experiments unless otherwise stated.
Randomization	All samples and animals were allocated in random.
Blinding	blinding was not possible in experiments since the same investigator performed the experiment and data analysis.

Reporting for specific materials, systems and methods

We require information from authors about some types of materials, experimental systems and methods used in many studies. Here, indicate whether each material, system or method listed is relevant to your study. If you are not sure if a list item applies to your research, read the appropriate section before selecting a response.

Materials & experimental systems

n/a	Involved in the study	
☐	☒	Antibodies
☐	☒	Eukaryotic cell lines
☒	☐	Palaeontology and archaeology
☐	☒	Animals and other organisms
☒	☐	Clinical data
☒	☐	Dual use research of concern

Methods

n/a	Involved in the study	
☒	☐	ChIP-seq
☐	☒	Flow cytometry
☒	☐	MRI-based neuroimaging

Antibodies

Antibodies used	Intagrin α2 (Abcam, ab133557), Intagrin β1 (Abcam, ab179471), NF-KB P65 (Cell Signaling Technology, 8242S), Phospho-NF-κB p65 (Ser536) (Cell Signaling Technology, 3033S), Slamf4 (Cell Signaling Technology, 54560S), anti-GST (Cell Signaling Technology, 2624S),
Validation	All antibodies are obtained from commercial sources, and vendors have shown validation on their websites.

Eukaryotic cell lines

Policy information about cell lines and Sex and Gender in Research

Cell line source(s)	Colon cancer cell lines HT-29 , Caco-2, HCT116, MC38 were obtained from ATCC. HT29 was isolated from a female patient. Caco-2 was isolated from a male patient. HCT116 was isolated from a male patient. MC38 was isolated from C57BL/6 mice. Colon normal immortalized epithelial cell line NCM460 was obtained from INCELL. NCM460 was isolated from the normal colon of a Hispanic male.
Authentication	The cell lines were bought from ATCC and INCELL with authentication (STR profiling).
Mycoplasma contamination	None of the cell lines were mycoplasma contamination
Commonly misidentified lines (See ICLAC register)	No commonly misidentified cell lines were used in this study.

Animals and other research organisms

Policy information about studies involving animals; ARRIVE guidelines recommended for reporting animal research, and Sex and Gender in Research

Laboratory animals	Description of research mice used for experiments can be found in the relevant figure legends and Methods. Mice were kept in specific pathogen-free facilities, following a 12-hour light/12-hour dark cycle, and had ad libitum access to food and water. Food and water were provided ad libitum.
Wild animals	This study did not involve wide animals.
Reporting on sex	Findings only applied to male mice.
Field-collected samples	This study did not involve field-collected samples.
Ethics oversight	All animal experiments were approved by the Animal Experimentation Ethics Committee of the Chinese University of Hong Kong

Note that full information on the approval of the study protocol must also be provided in the manuscript.

Flow Cytometry

Plots

Confirm that:

☒ The axis labels state the marker and fluorochrome used (e.g. CD4-FITC).

☒ The axis scales are clearly visible. Include numbers along axes only for bottom left plot of group (a 'group' is an analysis of identical markers).

☒ All plots are contour plots with outliers or pseudocolor plots.

☒ A numerical value for number of cells or percentage (with statistics) is provided.

Methodology

Sample preparation	Sample preparation was listed in Methods. colons were minced into pieces and incubated in a digestion buffer consisting of PBS, 1% BSA, 1 mg/mL collagenase type IV and 0.5mg/ml DNase I for 30 min at 37?. The cell suspensions were filtered through a 70µm strainer and then centrifuged at 2000 rpm, 10min. For surface marker staining, LP cells were stained with fluorescent-conjugated antibodies in cell staining buffer for 20 min. For intracellular staining, cells were stimulated for 4 h in RPMI complete medium with 30 ng/ml phorbol 12-myristate 13-acetate (Abcam) and 1µg/ml ml ionomycin (STEMCELL Technologies) in the presence of 2.5µg/ml monomycin (BioLegend). Then, cells were stained with cell surface markers. Samples for flowcytometry analysis were fixed with the cell fixation buffer (BioLegend), permeabilized with FOXP3 Perm buffer (eBioscience) according to the manufacturer's recommendations and stained with intracellular antibody.
Instrument	BD FACSAria Fusion
Software	BD FACSAria Fusion, Flowjo v9
Cell population abundance	Tumor infiltrating CD45 positive cells from each mice colon (50000-100000 cells) were used to further identify different kinds of immune cells by its surface markers.

Gating strategy

Gating strategy of lymphocytes from mice, including MDSCs (CD11b+Gr1hi), M-MDSC (CD11b+Gr1hiLy6ChiLy6G-), G-MDSC (CD11b+Gr1hiLy6CintLy6G+), pDCs (CD11C+ MHC-Π+CD11b-B220+), mDCs (CD11C+MHC-Π+CD11b+B220-), gd T cell (CD3 +TCR gd+), CD4+ T (CD3+CD4+), CD8+ T (CD3+CD8+), ILC1 (Lin1-Lin2-CD90.2+ST2-RORgt-), ILC2 (Lin1-Lin2-CD90.2+ST2 +RORrt-), ILC3 (Lin1-Lin2-CD90.2+ST2-RORrt+). Lin1: CD11c, CD5; Lin2: CD3, B220, F4/80, CD11b.

☒ Tick this box to confirm that a figure exemplifying the gating strategy is provided in the Supplementary Information.
